# Supplementary material for: Intratumoral Niches of B Cells and Follicular Helper T Cells, and the Absence of Regulatory T Cells, Associate with Longer Survival in Early-Stage Oral Tongue Cancer Patients
Source: Cancers (Basel). 2022 Sep 1;14(17):4298. doi: 10.3390/cancers14174298 (PMC9454508; doi:10.3390/cancers14174298)
Supplement: Supplementary file 1 [file cancers-14-04298-s001.zip › cancers-1842092-supplementary.pdf]

Supplementary methods

Image analysis using TME-Analyzer

The in-house developed phython interface was used to perform image analysis. This analysis consisted of five steps. (1) Foreground selection: images were thresholded using all channels to generate a foreground area that covers regions positive for all signals. (2) Tissue detection and segmentation: cytokeratin-positive and negative regions of the foreground were identified as tumor and stroma regions, respectively. For steps 1 and 2, regions that were deemed too small were excluded. (3) Nucleus detection and segmentation: Nucleus detection was performed using a machine learning based published methodology using the default “2D_versatile_fluo” method [ref: U. Schmidt, M. Weigert, C. Broaddus, G. Myers, Cell Detection with Star-convex Polygons, presented at the International Conference on Medical Image Computing and Computer-Assisted Intervention (MICCAI), Granada, Spain, September 2018.]. (4) Cell segmentation: Once identified, center of nuclei were used as seeds for Voronoi segmentation on Foreground mask for determining individual cell regions. (5) Phenotyping: fluorescent intensities for the channels that correspond to each marker were analyzed per cell.

Table S1. Primary and secondary antibodies used for multiplex staining [a].

Sequence in panel	1st Ab					Buffer	2ndAb	Fluorophore
	Ab	Clone	Conc.	Provider	Incu. time (minute)			
1	CD4	EP204	1:100	Cell Marque	30	AR9	Opal Polymer HRP Ms and Rb	Opal 520
2	CD20	L26	1:200	Cell Marque	30	AR6		Opal 650
3	FOXP3	234A/E7	1:200	Abcam	30	AR6		Opal 690
4	Tbet	4B410	1:25	eBioscience	60	AR6		Opal 570
5	CD21	EP3093	1:100	Cell marque	3	AR9		Opal 540
6	BCL6	GI191E	1:200	eBioscience	30	AR9		Opal 620
7	CK	AE1/AE3	1:200	Invitrogen	30	AR6	TSA Coumarin System; Biotin-HRP, and Streptavidin- HRP	Coumarin

[a] Table lists 7-color multuplex staining protocol. Abrreviations: Ab: Antibody; Conc: Concentration; Incu. time, Incubation time.

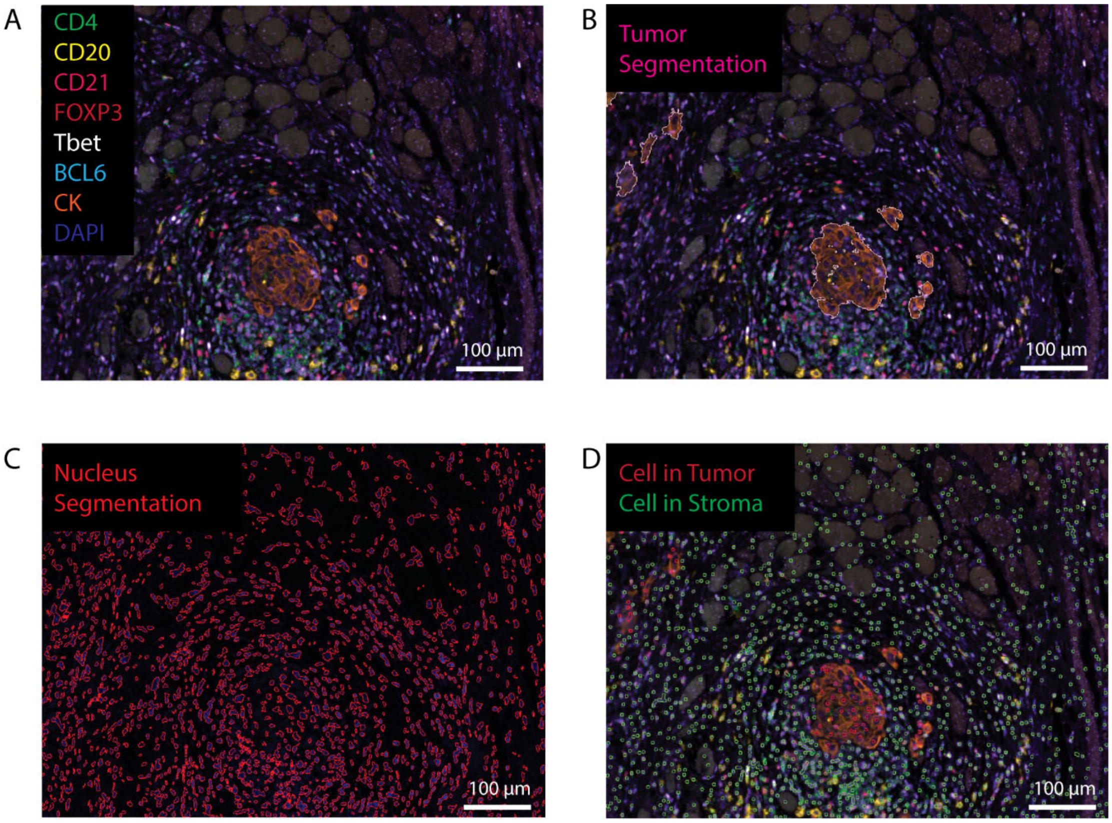

Figure S1. Example images for step-wise analysis using Tumor Microenvironment-Analyzer. (A) Representative multispectral images of immune cells in oral tongue cancer (20x magnification), and of the different steps, such as **(B)** tissue segmentation, **(C)** nucleus segmentation, and **(D)** overlay of nucleus location on tissue segmentation.

Variable		n	Survival	HR	p-value
**Univariate Cox analysis for OS**					
CD20 CS	Low	43	65.7		
	High	32	74.6	0.280	**0.025**
Gender	male	42	68.1		
	female	33	71.3	0.697	0.448
Age	<65	44	74.7		
	>=65	31	62.9	4.184	**0.006**
pT	1	38	73.5		
	2&3	37	65.5	2.058	0.130
pN	0	69	68.6		
	1	6	NA	0.043	0.370
pS	1	36	73.1		
	2&3	39	66.2	1.842	0.200
LRR	no LRR	65	70.0		
	LRR	10	66.6	1.212	0.760
TLS	Absence	15	72.0		
	Presence	60	69.0	0.948	0.925
**Multivariate Cox analysis for OS**					
CD20 CS				0.262	**0.019**
Age				4.405	**0.004**
pT				1.706	0.281

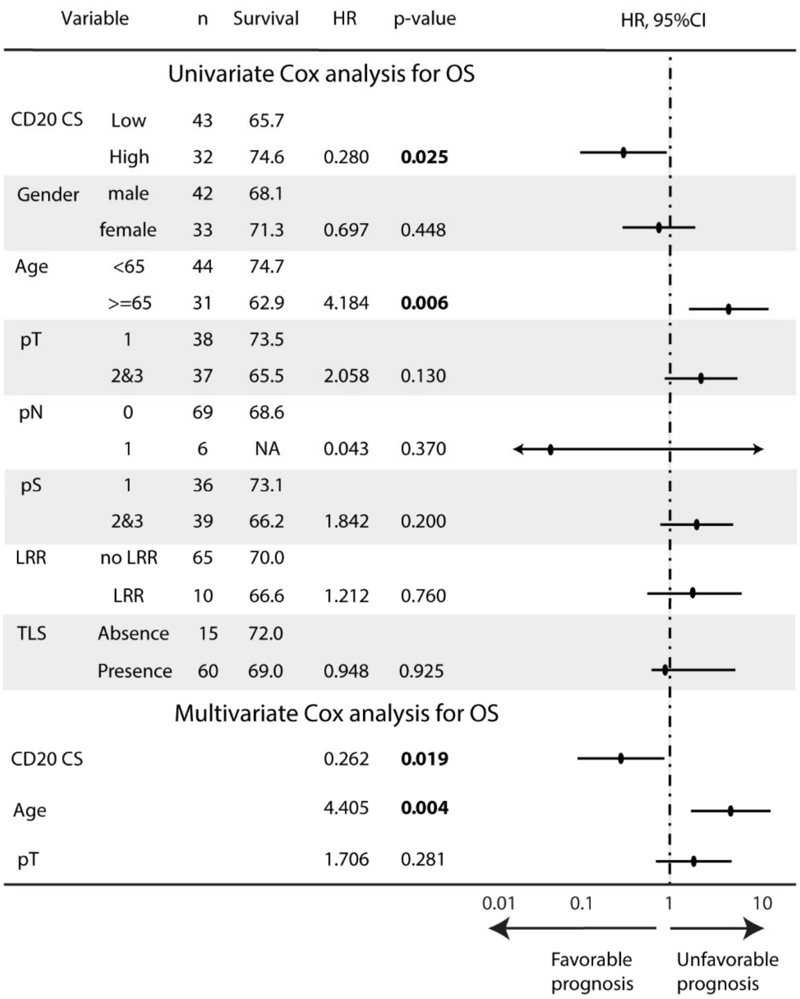

Figure S2. Univariate and Multivariate analysis of the prognostic value of the CD20 cluster score. Univariate and multivariate Cox proportional regression hazards models for OS. Estimated mean survival is shown for each variable. HR, 95% CI, and p-value are shown for both univariate and multivariate analysis; in case p-value < 0.05, this is highlighted in bold. Abbreviations: CD20 CS: CD20 cluster score; pT: pathological tumor stage; pN: pathological nodal stage; pStage: pathological stage; LRR: locoregional recurrence; TLS: tertiary lymphoid structure.

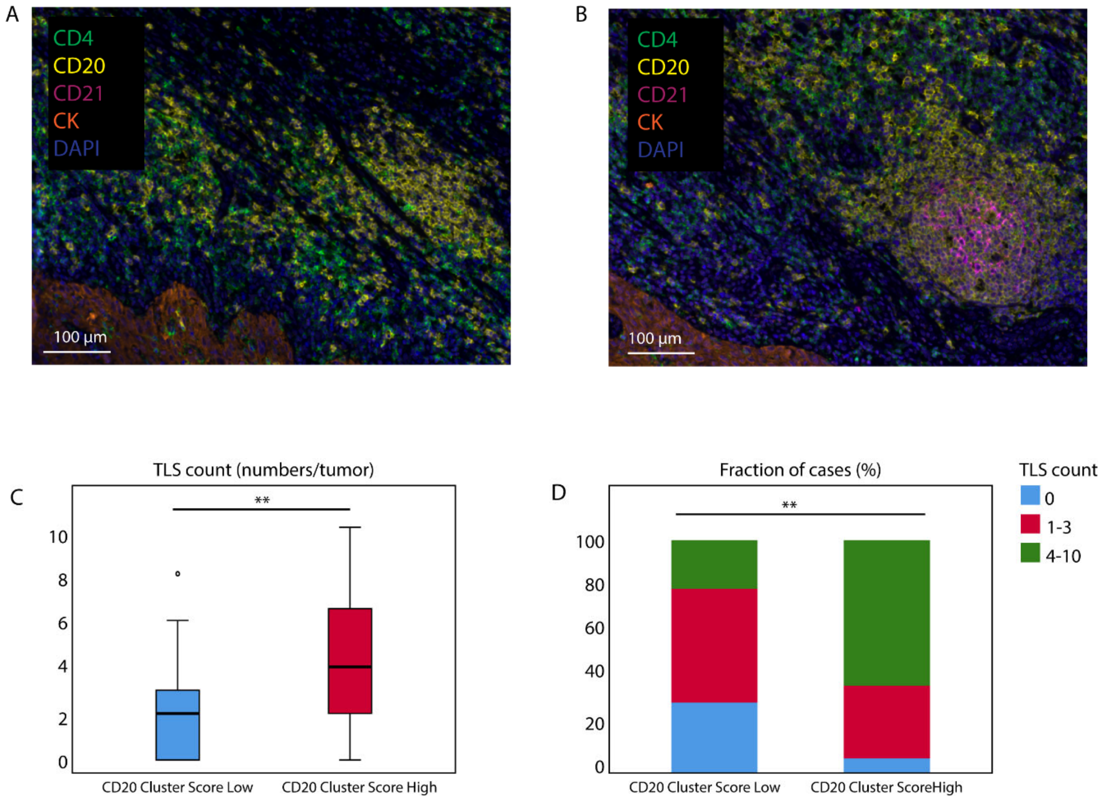

Figure S3. The CD20 cluster score associates with the TLS count. (**A, B**) Representative images of TLS from two CD20 cluster score high tumors with poor-organized zones of CD4 and CD20 cells (**A**, observed in the majority of tumors), and with highly dense presence of CD20 cells forming germinal centers together with CD21+ follicular dendritic cells (**B**, observed in the minority of tumors). (**C**) Boxplots showing TLS counts in patients with a high and low CD20 cluster scores, **: p-value <0.01 according to Mann-Whitney U test. (**D**) Stacked bars showing the fractions of different TLS count in patients with a high and low CD20 cluster scores, **: p-value <0.01 according to Chi-square test.

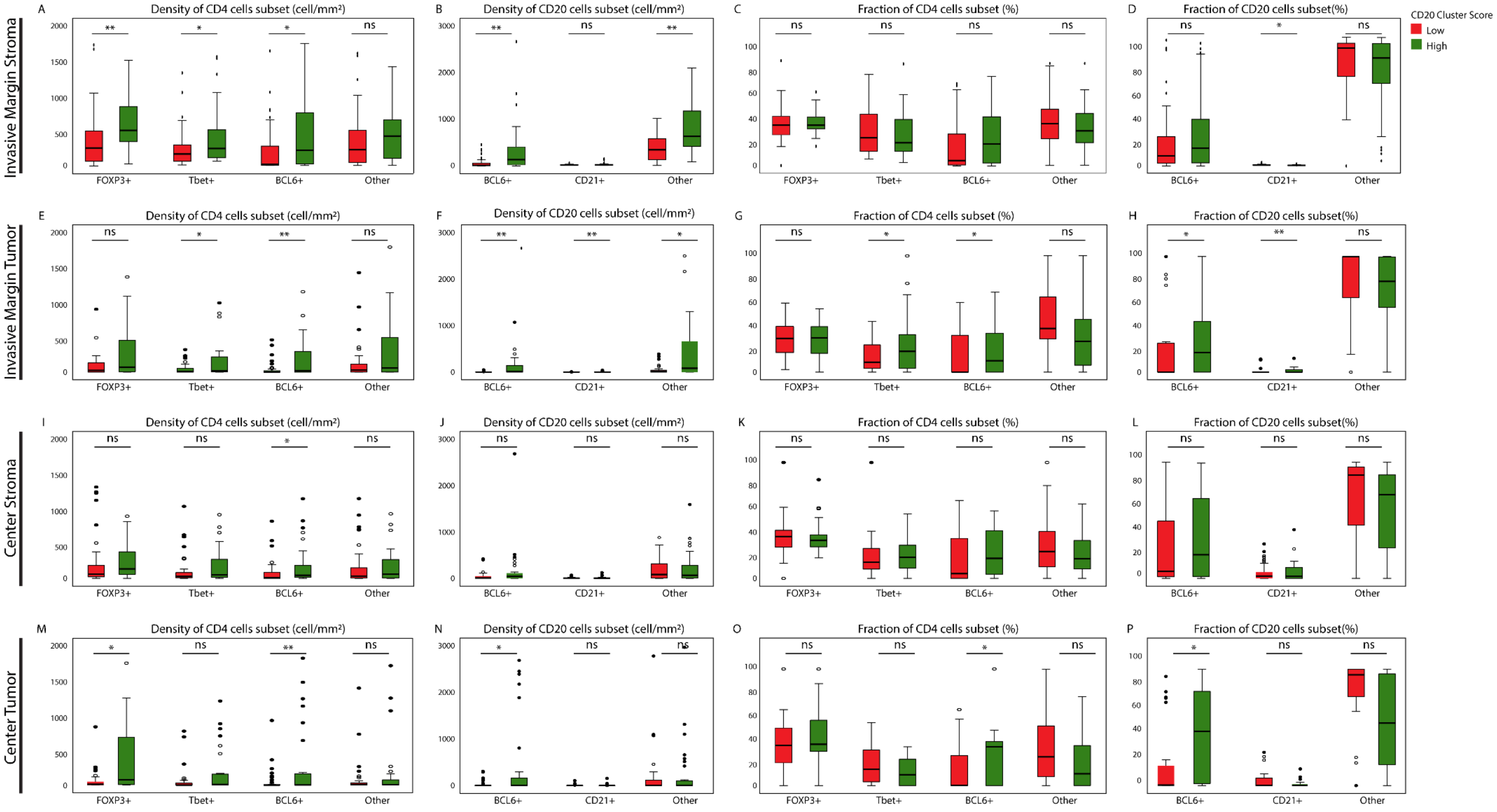

Figure S4. Density and fraction of CD4 and CD20 subsets according to the CD20 cluster score. (**A-P**) Boxplots showing density and fraction of CD4 and CD20 subsets at IM-S (**A to D**), IM-T (**E to H**), C-S (**I to L**), and C-T (**M to P**) regions. ns: not statistical significant, *: p <0.05, and **: <0.01 according to Mann-Whitney U test.

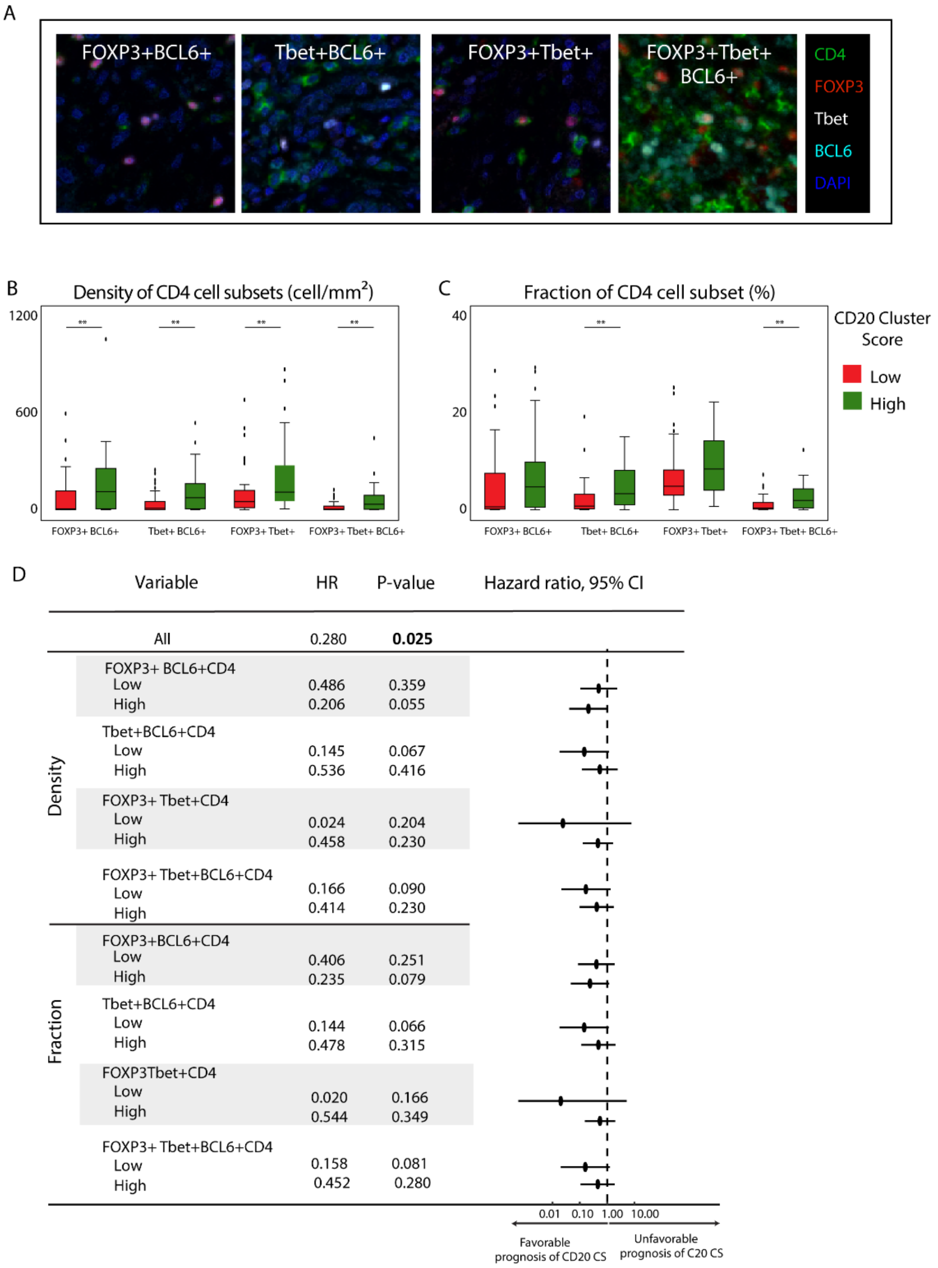

Figure S5. Presence of follicular regulatory T cells does not associate with the prognostic value of CD20 cluster score. (A) Representatives images of CD4 cells positive for more than one transcriptional factor: FOXP3+BCL6+ CD4+, Tbet+BCL6+ CD4+, FOXP3+Tbet+ CD4+, and FOXP3+Tbet+BCL6+ CD4+. (B-C) Box plots showing densities (B), and fractions (C) of the CD4 phenotypes from (A) according to the CD20 cluster score in invasive margin stroma (IM-S). Statistical significance according to the Mann-Whitney U test is shown above individual plots; **: $p<0.01$. (D) Forest plot of subgroup analysis for the CD20 cluster score according to density and fraction of the CD4 phenotypes from (A). It was not possible to quantify nearest neighbor interactions of CD4 phenotypes from (A) for a large portion of the cohort due to their scarcity. HR, 95% CI, and p-value are shown for each variable; in case p-value < 0.05, this is highlighted in bold. Abbreviations, CD20 CS: CD20 cluster score; HR: hazard ratio.

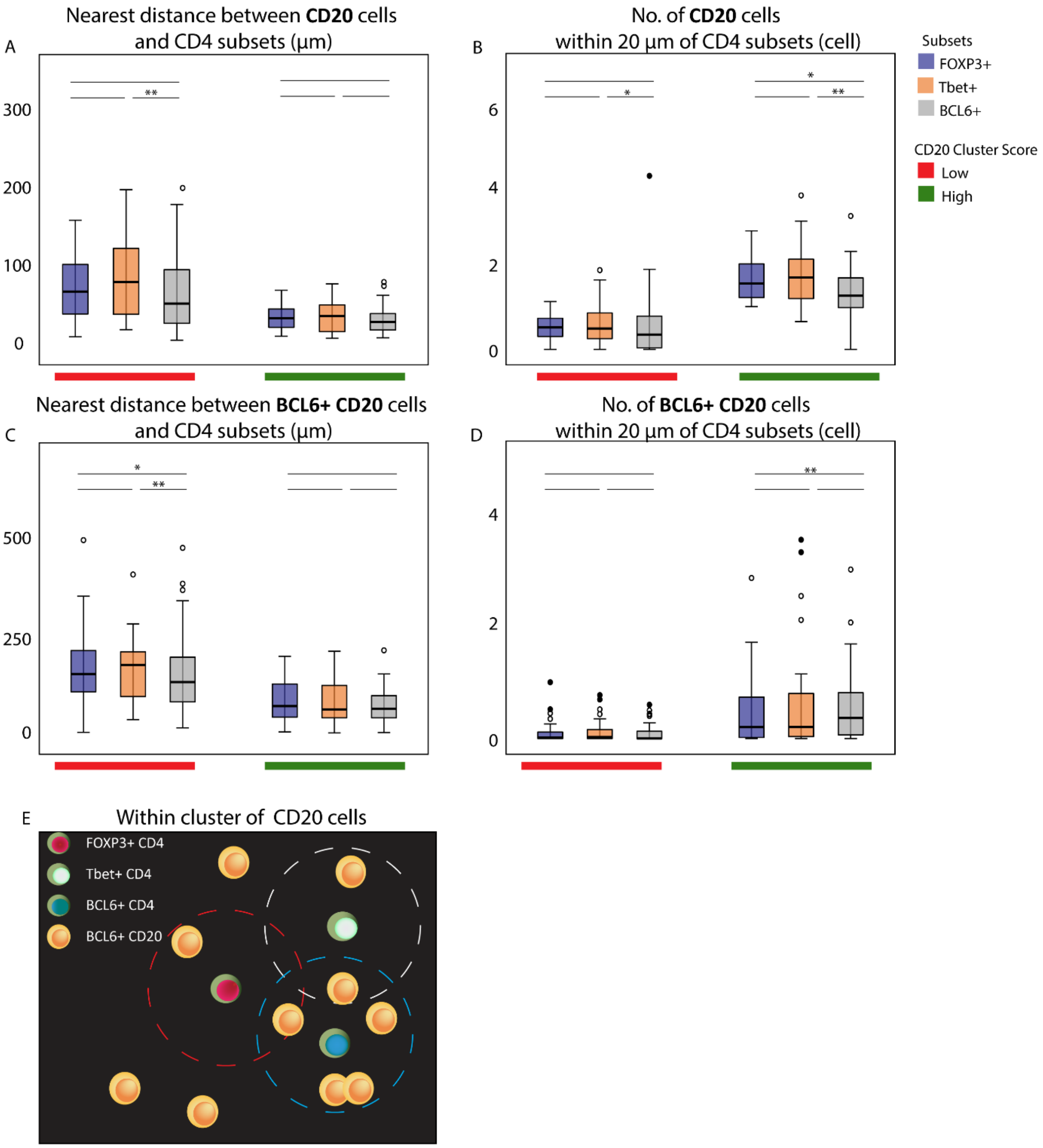

Figure S6. CD20 cluster score high tumors demonstrate GC B cells in close proximity of Tfh cells. (**A, B**) Boxplots showing nearest distance between CD20 and CD4 subsets (**A**), and number of CD20 cells within 20 µm of CD4 cells (**B**). (**C, D**) Boxplots showing nearest distance between BCL6+ CD20 and CD4 subsets (**C**), and number of BCL6+ CD20 cells within 20 µm of BCL6+ CD4 cells (**D**). *: p <0.05, and **: <0.01 according to Wilcoxon signed-rank test. (**E**) Cartoon illustrating analyses of number of BCL6+ B cells within radius of other subsets of CD4 cells.

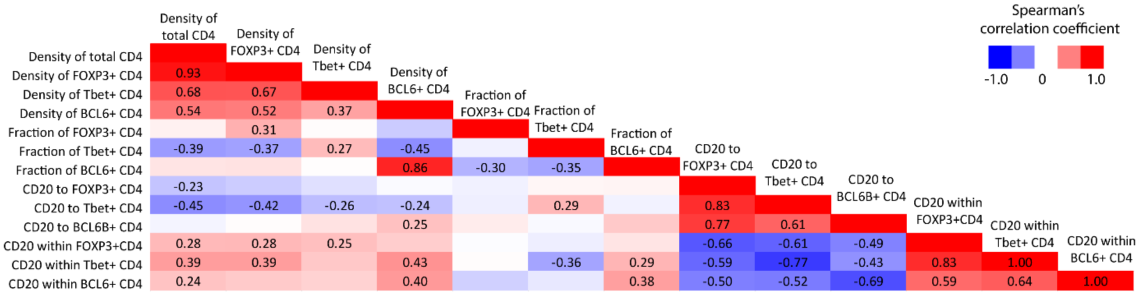

Figure S7. Fraction of Treg cells inversely correlates with that of Tfh cells in IM-S. Heatmap showing spearman's correlations between density of CD4 subsets, fraction of CD4 subsets, nearest distance between CD20 and CD4 subsets, and number of CD20 within 20 µm of CD4 subsets. Numbers within heatmap refer to the correlation coefficient in case statistically significant (i.e. p-value <0.05).

Variable	n	Estimated mean OS		HR	P-value
		CD20 CLS Low	CD20 CLS High		
**Density of CD20**					
Low	37	61.3	76.0	0.318	0.271
High	38	78.1	74.3	1.165	0.121
**Density of CD21**					
Low	37	57.9	70.0	0.232	0.066
High	38	72.9	78.4	0.388	0.075
**Density of BCL6+ CD20**					
Low	37	66.6	72.9	0.375	0.208
High	38	64.4	75.5	0.286	0.055
**Density of CD21+ CD20**					
Low	37	NA	NA	0.023	0.202
High	38	67.2	71.9	0.459	0.129
**Fraction of BCL6+ CD20**					
Low	38	67.8	74.4	0.318	0.151
High	37	62.3	74.7	0.256	0.053
**Fraction of CD21+ CD20**					
Low	38	65.7	76.6	0.142	0.077
High	37	66.0	70.5	0.592	0.162

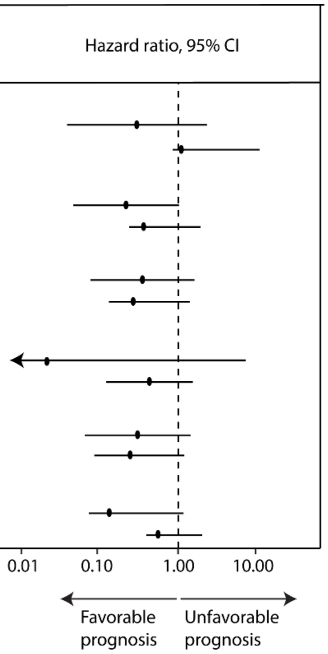

Figure S8. The CD20 cluster score is not affected by densities nor fractions of CD20 subsets. Forest plots of subgroup analysis of the CD20 cluster score for OS according to density and fraction of CD20 subsets. Estimated mean overall survival, HR, 95% CI, and p-value are shown for each variable. Abbreviations: CD20 CS: CD20 cluster score; HR: hazard ratio; NA: non-applicable.

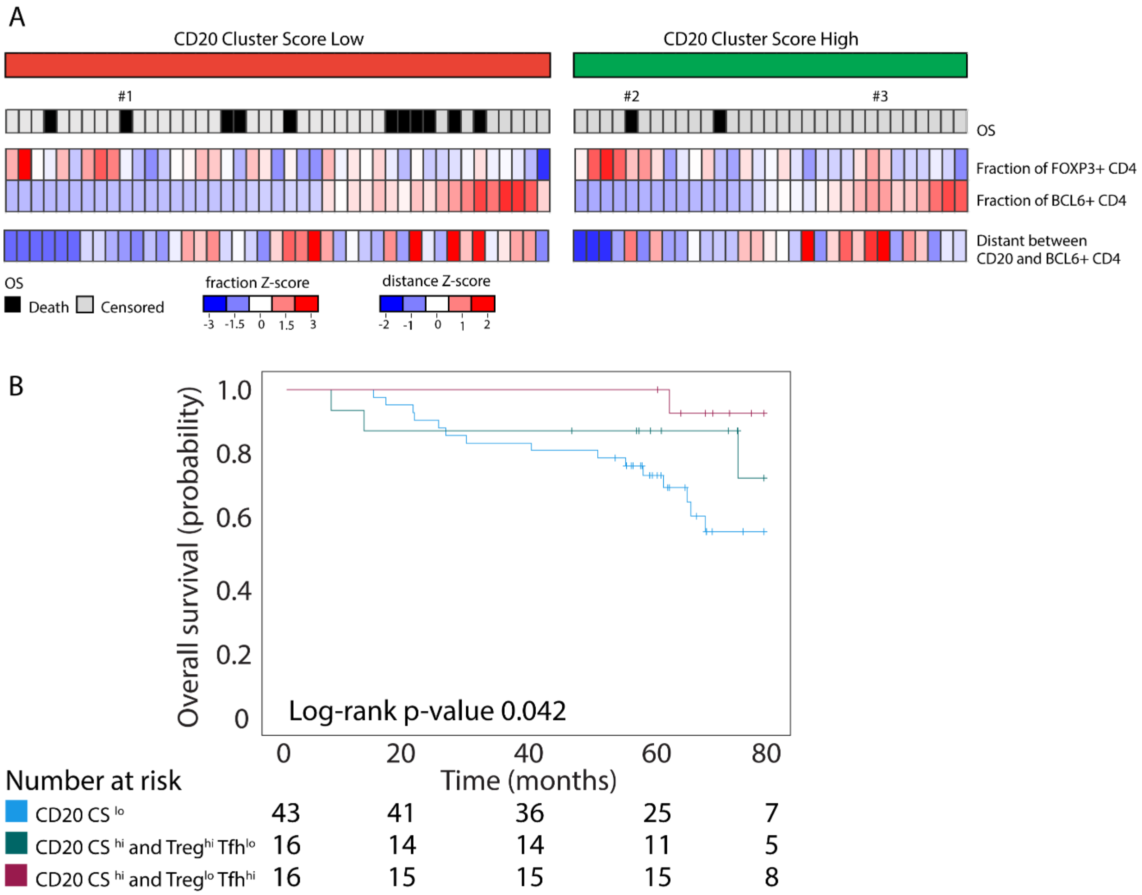

Figure S9. The CD20 cluster score together with the relative abundance of Tfh cells versus Treg cells provides maximal 5-yr overall survival. (**A**) Heatmap displaying the association between the CD20 cluster score and 5-year overall survival, and normalized Z-scores for fractions of Tfh and Treg cells in IM-S, and nearest distances between B cells and Tfh cells. Tumors are sorted based on their Tfh:Treg cells ratio. Numbered tumors correspond to **Figure 5**: #1 for a scenario where the CD20 cluster Score is low; #2 for a scenario where the CD20 cluster score is high and the relative abundance of Tfh versus Treg is low; and #3 for a scenario where the CD20 cluster score is high and the relative abundance of Tfh versus Treg is high. (B) Overall survival analysis of the CD20 cluster score together with the relative abundance of Tfh versus Treg cells. Abbreviation: CD20 CS, CD20 cluster score.
